# New Perspectives on Spontaneous Brain Activity: Dynamic Networks and Energy Matter

**DOI:** 10.3389/fnhum.2016.00247

**Published:** 2016-05-26

**Authors:** Arturo Tozzi, Marzieh Zare, April A. Benasich

**Affiliations:** ^1^Center for Nonlinear Science, University of North TexasDenton, TX, USA; ^2^Computational Intelligence Laboratory, University of ManitobaWinnipeg, Canada; ^3^School of Computer Science, Institute for Research in Fundamental Sciences (IPM)Tehran, Iran; ^4^Center for Molecular and Behavioral Neuroscience, Rutgers University-NewarkNewark, NJ, USA

**Keywords:** spontaneous activity, free energy principle, power law, phase transition, energetic constrains

## Abstract

Spontaneous brain activity has received increasing attention as demonstrated by the exponential rise in the number of published article on this topic over the last 30 years. Such “intrinsic” brain activity, generated in the absence of an explicit task, is frequently associated with resting-state or default-mode networks (DMN)s. The focus on characterizing spontaneous brain activity promises to shed new light on questions concerning the structural and functional architecture of the brain and how they are related to “mind”. However, many critical questions have yet to be addressed. In this review, we focus on a scarcely explored area, specifically the energetic requirements and constraints of spontaneous activity, taking into account both thermodynamical and informational perspectives. We argue that the “classical” definitions of spontaneous activity do not take into account an important feature, that is, the critical thermodynamic energetic differences between spontaneous and evoked brain activity. Spontaneous brain activity is associated with slower oscillations compared with evoked, task-related activity, hence it exhibits lower levels of enthalpy and “free-energy” (i.e., the energy that can be converted to do work), thus supporting noteworthy thermodynamic energetic differences between spontaneous and evoked brain activity. Increased spike frequency during evoked activity has a significant metabolic cost, consequently, brain functions traditionally associated with spontaneous activity, such as mind wandering, require less energy that other nervous activities. We also review recent empirical observations in neuroscience, in order to capture how spontaneous brain dynamics and mental function can be embedded in a non-linear dynamical framework, which considers nervous activity in terms of phase spaces, particle trajectories, random walks, attractors and/or paths at the edge of the chaos. This takes us from the thermodynamic free-energy, to the realm of “variational free-energy”, a theoretical construct pertaining to probability and information theory which allows explanation of unexplored features of spontaneous brain activity.

## Introduction

Neural oscillations are observed throughout the central nervous system (CNS) and at all levels. Here we are specifically concerned with “spontaneous brain activity”, that is brain activity generated in the absence of an explicit task and frequently correlated with “resting-state or default-network activity” (Raichle et al., 2001). Much of what is known concerning the intersection of brain anatomy and function comes from functional magnetic resonance imaging (fMRI) studies that highlight task-evoked responses. In general, fMRI studies focus on changes in the blood oxygenation level-dependent (BOLD) signal, which is induced by the neural response to an externally controlled stimulus/task (Damoiseaux et al., 2006). These experiments, as well as those employing a variety of other techniques, encourage the view that two different types of neuronal activity drive brain function: evoked and spontaneous. However, such an approach ignores an alternative possibility: that the brain’s operations are *primarily spontaneous* and involve the acquisition and maintenance of information (Raichle, 2010; Barrett and Simmons, 2015). Multiple techniques, such as fMRI, electroencephalography (EEG) and magnetoencephalography (MEG), show that spontaneous, low-frequency fluctuations of cerebral activity, which cannot be attributed to the experimental design or other explicit input or output (Fox and Raichle, 2007), are temporally coherent within distributed, spatially independent, functional networks, and resemble those evoked by sensory, motor, and cognitive paradigms (de Pasquale and Marzetti, 2014). Spontaneous activity of the brain, first formally characterized 30 years ago, has received growing attention, as witnessed by the ever-increasing number of articles dedicated to this topic (Fox and Raichle, 2007; Buckner et al., 2008). Although sceptical researchers hold that BOLD signals mainly originate from cerebral blood flow (Tong and Frederick, 2010), it is widely believed that spontaneous signals are best correlated with spikes in the range of slow cortical potentials (Raichle et al., 2001). Indeed, spontaneous fluctuations have not only been observed in electric activity, but also in various hemodynamic and metabolic parameters, including spontaneous fluctuations in the membrane potential, spontaneous spikes and neurotransmitter release (O’Donnell and van Rossum, 2014). Multiple observations have documented the finding that fluctuations in spontaneous neurotransmitter release show a 1/f frequency distribution, similar to that of BOLD. Recent reports also suggest that such spontaneous neurotransmission release events have an autonomous role in intra-neuronal communication—as well as in the regulation of synaptic plasticity and homeostasis—distinct from that of evoked release and independent of presynaptic action potentials (Kavalali, 2015). Such findings highlight the realization that spontaneous brain activity has a vital functional role, is produced at all levels of the brain, and can, with the appropriate analytic and theoretical approaches provide unparalleled insights into dynamic brain function.

This review comprises five sections in which we hope to address this rapidly expanding area of research. The first section highlights the conceptual view that spontaneous fluctuations are the basic, standard architecture, not just of functional, but also of anatomical brain organization. Section two describes the high energetic requirements of the brain, but also emphasizes the fact that “spontaneous activity” requires *less* energy than “evoked activity”. Starting from such an assumption, the following section aims to explain spontaneous activity using the framework of “energetic landscapes” and complex, nonlinear systems. Section four reviews the most recent experimental studies, which have explored the link between spontaneous brain activity and psychological states. The fifth section, starting from the ubiquitous phenomenon of power laws and its correlations, provides an overview of one of the most successful general brain frameworks, Friston’s (2010) “Free Energy Principle”, in order to explore the power of this theory to explain the role of spontaneous brain activity.

## Spontaneous Activity is the Intrinsic Architecture of Brain Organization

It is clear that spontaneous brain activity cannot be simply reduced to “background noise” uncorrelated to the system response, rather it occurs during unconstrained “resting states”; that is, in subjects who are lying quietly with eyes closed, or while fixating on a target, but with no explicit task instruction, directed to think of nothing in particular, but importantly, awake. Spontaneous signals detected and recorded by fMRI, EEG or MEG support the hypothesis that spontaneous fluctuations constitute the basic architecture of functional brain organization (Cole et al., 2014). Spontaneous cortical electric activity is already present in the foetus by the 34th week of gestation (Krueger and Garvan, 2014); the brain spontaneously pulsates during the fatal period, in the same manner as the heart spontaneously contracts. It has also been demonstrated that spontaneous fluctuations are more frequently observed in immature synapses as compared to those that are more mature (Kavalali et al., 2011). In addition, the vertebrate spinal cord and brainstem possess central pattern generator (CPG) circuits, which can produce meaningful functional output in the absence of sensory inputs. Neocortical circuits could be regarded as particularly plastic types of CPGs, as they have rich spontaneous dynamics that are powerfully modulated or engaged by sensory inputs, but can also generate output in their absence (Yuste et al., 2005). Recent results also demonstrate that gamma-band activity in the alert monkey is largely an emergent property of cortex, arising from the resting state waves (Bastos et al., 2014). These fluctuations in cortical excitability exert a remarkable effect on other elements of the field potential’s activity, as well as on the spiking activity of neurons. In fact, this “coupling” or nesting of many spikes subserves an important coordinating role and provides a logical structure for the integration of functional activity (Buszáki, 2006). In sum, spontaneous fluctuations appear to represent the standard architecture of the nervous system’s functional organization (Cole et al., 2014), have a critical functional role, are produced by brain circuits and provide a window into their dynamic operations.

Spontaneous activity has been shown to correlate with brain function, as well as with precise anatomical structures. Analyses of spontaneous fluctuations within fMRI signals have identified a number of large-scale intrinsic networks, that is, regions with similar functionality and synchronous activity, which tend to be dynamically correlated in their spontaneous BOLD activity. Current data support the hypothesis that resting state networks have long-term stability, but in very flexible configurations (Gonzalez-Castillo et al., 2014). Among the networks exhibiting coherent fluctuations in spontaneous activity during rest, the “default-mode network” (DMN) is of particular interest. The DMN includes functionally and structurally connected regions that show high metabolic activity and blood flow at rest, but deactivate when specific goal-directed behavior or cognition is needed (Raichle et al., 2001). Despite recent, still controversial claims (Vatansever et al., 2015), a feature of the DMN is the inverse relationship of its neuronal activity with another intrinsic network, namely the “attentional network”. The attentional network is activated during externally-directed cognition and is engaged during cognitive tasks that require focused attention; importantly, it is deactivated during internally-directed cognition, whereas the opposite is true of the DMN.

## Energetic Requirements of Spontaneous Activity

The brain’s energy consumption follows the traditional thermodynamic formula:

E=G+TS

where, *E* is the enthalpy, *G* is the free-energy, *T* is the temperature and *S* is the thermodynamic entropy. Note that the formula, contrary to our simplified account, does not talk about absolute values, but of their variation. In simpler words, enthalpy represents the “fuel” coming into the brain. Enthalpy grossly corresponds to the sum of the “useful” or “free-energy” (i.e., the energy that can be converted to do work) and energy dissipation (e.g., entropy).

The brain represents 2% of the human body mass yet it accounts for about 20% of total energy consumed (corresponding to the enthalpy E), a very substantial proportion by any measure (Raichle et al., 2001; Fox and Raichle, 2007). The metabolic activity of the brain, influenced by a balance between the energy costs incurred by its operation and the benefits realized by energy expenditure, is therefore high and remarkably constant over time (Sengupta et al., 2013). Why does the brain consume such remarkable amounts of enthalpy, despite the fact that evolution is geared toward minimizing very high metabolic costs? Almost 20–60% of the enthalpy allocated for the brain is used to support the specific metabolic rate of the cortical gray matter, primarily the restoration of transmembrane potentials, and for neuronal signaling purposes—e.g., action potentials and synapses (Sengupta et al., 2013). Although the brain as a whole consumes large amounts of enthalpy, there are important differences between the total energy required for spontaneous vs. evoked neural activity. Evoked activity (i.e., perceptual and motor activity, task performance and similar cognitive functions), requires an additional energy consumption of ~5%, as compared with spontaneous activity. This is due to local increases in spike frequency (in particular beta and gamma waves) during evoked activity, which causes a transitory increase of energy consumption and free-energy production. A key point here is that increased spike frequency during evoked activity has a significant metabolic cost, specifically 6.5 μmol/ATP/gr/min for each additional spike, with an ATP consumption higher than the “typical” mean rate at 4 Hz (which is 3.29 × 10^9^ molecules of ATP/neuron/s; Attwell and Laughlin, 2001). These observations strongly suggest that, although spontaneous brain activity consumes a significant proportion of the brain’s vast energy budget (the enthalpy used by various neuronal processes produces an average of about four spikes/s), that amount is appreciably less than the more energy-intensive evoked activity (Attwell and Laughlin, 2001; Sengupta et al., 2013), Thus, it is clear that spontaneous brain activity exhibits a lower energetic level as compared with evoked activity. In sum, the fact that spontaneous brain energy has a lower energy level than evoked activity is clear in terms of spikes. Indeed, we know that each spike has a certain consumption of ATP. Each spike is formed by an oscillation, equipped with both amplitude and frequency. For Ohm’s law, energy consumption due to the AMPLITUDE of the oscillation is negligible as compared with energy consumption due to the FREQUENCY of the oscillation. This means that evoked activity, equipped with a mean frequency of spikes higher than spontaneous activity, expends more energy, both in terms of enthalpy and free-energy. In the following sections, this noteworthy statement allows us to analyze how the complexity of such an adaptive system is best understood as a “dynamic network” that aims to decrease its free-energy via entropy transfer.

## Under Spontaneous Activity, the Brain Exhibits Complex Dynamics

Recent models of nervous activity are starting to incorporate the complexity of adaptive evolving systems as dynamic relationship networks (Fraiman and Chialvo, 2012). Indeed, brain function can be analyzed not only through old-fashioned cause/effect, linear paradigms typical of deterministic systems, but in other, more promising ways. The concept of a nonlinear brain needs to be framed into the energy landscape theory, a concept built for assessment of potential surfaces in proteins (Bryngelson and Wolynes, 1987; Tozzi et al., 2016). In such a framework, the brain stands for a “phase space”, an abstract space where particle trajectories travel. In line with recent “nonlinear” neuronal theories (Friston and Ao, 2012), each brain function can be mathematically treated as a particle that crosses abstract, functional landscapes made of energy valleys, peaks and basins. Whirlpools (called attractors) are located within these landscapes and correspond to low-energy basins, which transport the particles (Watanabe et al., 2014). The particle movements occur through transitory, erratic, stochastic processes (called “random walks”), giving rise to self-organized, self-assembled and self-sustained structures (Bak et al., 1987; Strogatz, 2001; Vuksanovic and Hövel, 2014). Such nonlinear techniques are able to predict the evolution of trajectories in time; for each of the cycles around the attractor, there is a characteristic nonlinear scaling law (Newman, 2005) that governs the amplitude and period of the orbit and that can be quantified via differential equations. Among the several models described in the literature, the fixed-point attractor—a funnel-like location in phase space where trajectories converge as time progresses, following the shortest path—is suggested by recent articles (Sengupta et al., 2016; Tozzi et al., 2016). However, several alternative neuronal models to fixed-point attractors have been proposed: for example, it has been suggested that brain function exhibits a stable sequence, called transient heteroclinic channel (Afraimovich et al., 2013) and that, at least during spontaneous brain activity, the brain might display a functional torus (Tozzi and Peters, 2016). Further, concepts like communication-through-coherence (Fries, 2005; Deco and Jirsa, 2012) must also be taken into account.

Apart from nonlinearity, brain activity retains the characteristics of a complex system with non-equilibrium dynamics. This means that a large number of interacting and inter-dependent components exhibit emergent properties (i.e., properties that cannot be found at the micro-level state of organization, but are typical of the macro-level state). Changes propagate through interlinked levels, inducing connectivity and interaction at all scales of the brain system. Thus cognitive processes become observable products of the underlying dynamical system, with macroscopic features emerging from the sum of high fluctuating elements. Accordingly, behavior emerges as a macroscopic property from the renormalization of microscopic brain events (Papo, 2014).

It has also been proposed that the brain operates near criticality (Beggs and Timme, 2012) at the edge of chaos. That is, it works within a narrow window between randomness and regularity, forming a functional regime where information processing occurs as rapidly as possible (Tognoli and Kelso, 2014). It has been suggested that the brain exists near a second-order phase transition (Afraimovich et al., 2013), a state characterized by peculiar dynamical features—such as the universal power laws described in the next paragraphs. Claims of brain criticality are still quite controversial (Beggs and Timme, 2012). For example, Priesemann et al. (2014) suggest that neural activity does not reflect a self-organized critical state, but a slightly sub-critical regime. Other researchers suggest that brain function does not exhibit erratic brain dynamics nor attractors, but rather exhibits a stable sequence (Afraimovich et al., 2013).

Another possibility is that the brain could be in phase transition during spontaneous activity, but be out-of-phase during evoked activity (i.e., while processing stimuli coming from the external world). Indeed, under spontaneous conditions, the CNS displays the slow dynamics associated with low free energy (Papo, 2014). This suggests that spontaneous brain activity might lie at the bottom of the energy landscapes’ deep basins, in steep valleys where the free energy is (relatively) lower, as compared with the peaks. In such valleys, various dynamical phenomena might counteract the dissipation of brain energy. In particular, attractors could contribute to slowing the loss of energy and decreasing the entropy production typical of random systems (Gaspard, 2005). During spontaneous fluctuations, energy expenditure is thus balanced by homeostatic mechanisms—such as attractors and power laws (Vuksanovic and Hövel, 2014), in an effort to minimize free-energy. It has been proposed that, in the presence of attractors, resting-state networks emerge as “structured noise fluctuations” around a stable, low-firing equilibrium state (Deco and Jirsa, 2012). In contrast to spontaneous brain activity during rest, engaging in effortful cognitive tasks seems to modify brain function in a different manner. Neural systems can evolve from a subcritical regime of randomness to a critical state and then, beyond the critical value, to a supercritical regime, characterized by regularity and absence of complexity (Zare and Grigolini, 2013; Papo, 2014). This raises the possibility that evoked activity could lead to an exit from the phase transition that is typical of spontaneous activity. In sum, the spontaneous activity of the brain is an example of an “open system”, partly stochastic due to intrinsic fluctuations, that maintains an interplay between structural bottlenecks and non-equilibrium steady-state dynamics (i.e., homoeostasis or allostasis), in the face of environmental fluctuations (Friston, 2010).

## Mental Functions and Spontaneous Activity

There is ongoing discussion as to whether spontaneous fluctuations reflect changes of the underlying brain physiology independent of neuronal function, or instead reflect the neuronal baseline activity of the brain, when goal-directed neuronal action and external input are absent (Damoiseaux et al., 2006). Despite some claims to the contrary (Fox et al., 2015; Vatansever et al., 2015), it is widely believed that intrinsic activity is associated with unconstrained, conscious cognition (i.e., mind-wandering or day dreaming propensities; Kucyi and Davis, 2014) and with higher cognitive functions, such as internally-oriented cognition, autobiographical memory (Conway and Pleydell-Pearce, 2000; Morewedge et al., 2014), self-referential processing, affective decision making and autobiographical memory (Philippi et al., 2015). Others hypothesize that spontaneous functional connectivity patterns at rest might well constitute a “signature of consciousness”, reflecting a continuous stream of ongoing cognitive processes as well as random fluctuations shaped by a fixed anatomical connectivity matrix (Barttfeld et al., 2015). It has also been recently proposed that the DMN *is* the dreaming state (Domhoff and Fox, 2015).

Different DMN subsystems play an important role in aspects of internally-directed, spontaneous self-generated thought (characterized by their independence from external stimuli), such as accessing conceptual knowledge or autobiographical memories, conceptual processing, experiences focused on the future, construction of coherent mental scenes, retrieval of information related to self and other, to be reflected upon in a meta-cognitive manner (for a description of the terminology, see Andrews-Hanna et al., 2014) and open-monitoring meditation (Marzetti et al., 2014). Recent evidence suggests considerable overlap between the DMN and regions involved in self- and other-related mental processes—such as social, affective and introspective processes—(Amft et al., 2015). In line with the celebrated experiments of Libet et al. (1983), our ability to make choices might arise from random fluctuations in the brain’s background electrical noise; this suggests that individuals might well behave independently of cause and effect (Bengson et al., 2014).

If the above described brain functions are truly correlated with spontaneous activity, it means that they display lesser energetic requirements that other activities, such as sensation or perception. This testable proposition is an example of how our hypothesis about the lower energetic expenditure of spontaneous activity might be empirically proved. Some evidence already exists: e.g., it has been demonstrated that, during mind wandering, sensations and perceptions entrain the brain in a state of lower energy expenditure (Fox et al., 2015). In terms of the dynamical systems theory framework, this might correspond to the basins of the spontaneous networks, which form a temporary random walk for spontaneous thoughts.

In the general context of the brain as a whole, spontaneous activity provides a bridge that links different actors that come into play, for example among anatomic structures, energetic requirements and functional connectivity. Indeed, the low-energy activity constitutes such a conceptual bridge, because it exhibits both anatomical/functional (spontaneous brain activity and DMN) and psychological correlates (spontaneous, deliberate, self-generated thoughts; Andrews-Hanna et al., 2014).

## Free-Energy Theory and Spontaneous Brain Activity

The brain activity observed at many spatiotemporal scales exhibits a 1/f^n^-like power spectrum (Newman, 2005), including not just macroscopic electric oscillations, EEG, MEG and fMRI signals (He, 2014), but also microscopic membrane potentials and fluctuations in neurotransmitter release (Linkenkaer-Hansen et al., 2001; Milstein et al., 2009). In particular, the frequency spectrum of cerebral electric activity displays a scale-invariant behavior S(f) = 1/f^n^, where S(f) is the power spectrum, f is the frequency and *n* is an exponent that equals the negative slope of the line in a log power vs. log frequency plot (Pritchard, 1992; Van de Ville et al., 2010). Pink noise has been regarded as an intrinsic property of the brain characterizing a large class of neuronal processes (de Arcangelis and Herrmann, 2010); moreover, power law distributions contain information about how large-scale physiological and pathological outcomes (Jirsa et al., 2014) arise from the interactions of many small-scale processes. The emergence of power law distributions in the brain (i.e., the spontaneous neuronal “avalanches” occurring in the upper cortical layers) has been interpreted in terms of self-organized criticality (Beggs and Plenz, 2003); thus, spontaneous brain activity has been proposed as a source of ideal 1/f noise (Allegrini et al., 2009).

In addition, the connectome displays a free-scale structure, involving not just temporal power laws, but also geometric fractals (Reese et al., 2012): this interesting observation further supports the hypothesis that the brain is in phase transition. However, the presence of power-law scalings in the brain remains controversial (Beggs and Timme, 2012). It must be emphasized that the fractal slope is not invariant in brain, but is rather characterized by multiple possible exponents (He et al., 2010), summarized by a single value, the “generalized fractal dimension”. Accordingly, we may view the multifractal cortex as an ensemble of intertwined (mono)fractals, each with its own dimension and scaling slope: the brain is thus regarded as a system of fractal geometry with a complex spectrum of self-exact similarity breakdown, in which scaling exponents mark dynamical transitions between different response regimes (Papo, 2014). With an increase in free-energy, the power law exponent varies across brain regions (Pritchard, 1992; He et al., 2010; Beggs and Timme, 2012; Tinker and Velazquez, 2014). The question that arises is: do cortical fluctuations in power law exponents modify the energy of the system? And the answer is yes. Recent articles have begun to uncover connections between such an exponent and activation free-energy, specifically in escape paths from energy basins (Perkins et al., 2014). Furthermore, it has been shown that fractals ensure an increase of production of entropy, when a system is close to equilibrium (Gilbert et al., 2000). The critical slowing implicit in power law scaling is mandated by any system that tries to minimize its energetic expenditure. For example, it has been demonstrated that brain regions with a larger fractal exponent are more expensive in their glucose metabolism (He, 2011).

Overall then, ongoing fluctuations with complex scale-free properties, such as spontaneous brain oscillations, can be absorbed into a free-energy principle (Friston, 2010), a theoretical framework that is of central importance in this context. In such a framework, a general theory of spontaneous brain function arises in a biologically informed fashion and has the potential to be operationalized and empirically assessed (Barrett and Simmons, 2015; Papadopoulou et al., 2015). The free-energy principle for adaptive systems (that is, biological agents, like the brain) attempts to provide a unified account of action, perception and learning. Any self-organizing system at equilibrium with its environment must minimize its free-energy, thus resisting a natural tendency to disorder/entropy. This formulation reduces the physiology of biological systems to their homeostasis, specifically, the maintenance of their states and form in the face of a constantly changing environment.

The brain operates at the edge of a delicate equilibrium and therefore appears to avoid minimizing its thermodynamic entropy production. It corresponds to the principle of minimizing the so-called “variational free-energy” at each point in time. The concept of variational free-energy is slightly different from the above mentioned “classical” thermodynamical free-energy, because the former refers to Helmholtz free-energy, which is a functional of some outcomes and a probability density over their (hidden) causes. Such a tenet supports the attempt to understand how subtle steady-state equilibrium is maintained through apparent resistance to the natural tendency to increase entropy, in order that the living beings restrict themselves to a limited number of states. The variational free-energy construct is an information theoretic quantity, as opposed to a thermodynamic quantity, thus it has been exploited in machine learning and statistics to solve many inference and learning problems. (Friston, 2010). In this context, the “time average” of variational free-energy becomes a proxy for entropy.

From the point of view of the brain, the environment includes both the external and the internal milieu. The probability of sensory states (interoceptive and exteroceptive) must have low entropy and because entropy is also the average of self-information or “surprise”, the brain implicitly avoids surprises (Friston, 2010). The free-energy principle separates the environment (the external states) from the agent (the internal states). Agents suppress free energy (or surprise) via changing sensory input by acting on external states, or by modifying their internal states through perception. This embodied exchange with the environment endeavors to maintain, as far as possible, an unsteady state that is the essence of survival and self-organization. In the framework of a logical-mathematical formalism, the variational free energy considered above pertains to probability and information theory—but what role does thermodynamic free-energy play? Crucially, minimizing variational free-energy necessarily entails a metabolically efficient encoding that is consistent with the principles of minimum redundancy and maximum information transfer (Picard and Friston, 2014). Maximizing mutual information and minimizing metabolic costs are two sides of the same coin; by decomposing variational free energy into accuracy and complexity, one can derive the principle of maximum mutual information as a special case of maximizing accuracy, while minimizing complexity translates into minimizing metabolic costs (Friston et al., 2015). Thus, the basic form of Friston’s free-energy principle supports the idea that the energetic levels of spontaneous brain activity, which are lower when compared with evoked activity, allow the CNS to obtain two apparent contradictory achievements: to minimize as much as possible the metabolic costs, and to the largest extent possible, maximize mutual information.

In conclusion, every psychical state in living beings is a result of the self-preservative processes of the organism, a biological fact of the drive to maintain, as far as possible, a state close to a (more or less stable) equilibrium. This optimal point, around which the life of the organism moves in constant oscillation, also has a logical-mathematical significance when framed within an energetic theory of spontaneous brain activity. Thus, consideration of the energetic differences between spontaneous and evoked brain activity and the effect on dynamic cortical networks, when viewed using a non-linear dynamical framework, constitutes a new approach to better understanding spontaneous brain dynamics as a conceptual bridge between anatomical/functional and psychological correlates.

## Author Contributions

All authors had full access to all the data in the study and take responsibility for the integrity of the data and the accuracy of the data analysis. AT, MZ, AAB: Study concept and design. AT, MZ: Acquisition of data. AT, MZ, AAB: Analysis and interpretation of data. AT, MZ, AAB: Drafting of the manuscript. AAB: Critical revision of the manuscript for important intellectual content. AT, MZ, AAB: Statistical analysis. AAB: Obtained funding. AT, MZ, AAB: Administrative, technical, and material support. AT, MZ, AAB: Study supervision.

## Conflict of Interest Statement

The authors declare that the research was conducted in the absence of any commercial or financial relationships that could be construed as a potential conflict of interest.
